# Rescue of mitochondrial function in **parkin*-*mutant fibroblasts using drug loaded PMPC-PDPA polymersomes and tubular polymersomes

**DOI:** 10.1016/j.neulet.2016.06.065

**Published:** 2016-09-06

**Authors:** G. Yealland, G. Battaglia, O. Bandmann, H Mortiboys

**Affiliations:** a Biomedical Sciences, University of Sheffield, Firth Court, Western Bank, Sheffield S10 2TN, United Kingdom; b SITraN, Neuroscience, University of Sheffield, 385a Glossop Road, Sheffield S10 2HQ, United Kingdom; c Department of Chemistry, UCL, 20 Gordon Street, London WC1 H 0AJ, United Kingdom

**Keywords:** UA, ursolic acid, UCA, ursocholanic acid, PMPC-PDPA, poly(2-(methacryloyloxy)ethylphosphorylcholine)–poly(2-diisopropylamino)ethylmethacrylate), ATP, adenosine tri-phosphate, DMSO, dimethyl-sulfoxide, PD, Parkinson’s disease, UDCA, ursodeoxycholic acid, BBB, blood brain barrier, FDA, federal drug administration, MW, molecular weight, DHE, dehydroergosterol, DLS, dynamic light scattering, TEM, transmission electron microscopy, TFR, thin-film rehydration, HFF, hollow fibre filtration, RCF, relative centrifugal force, RP-HPLC, reverse-phase high performance liquid chromatography, TFA, trifluoroacetic acid, ELSD, evaporative light-scattering detector, SD, standard deviation, FBS, foetal bovine serum, MEM, minimum essential media, DMEM, Dulbecco’s modified eagle medium, TMRM, tetramethylrhodamine, methyl ester, perchlorate, MMP, mitochondrial membrane potential, LDH, lactate dehydrogenase, FSC, forward scattering, SSC, Sidescattering, ANOVA, analysis of variance, mPEG-PCL, methoxy-poly(ethylene glycol)–polycaprolactone, Akt, protein kinase B, Parkinson’s disease, Mitochondria, Parkin, Polymersome, Anisotropic nanoparticle, PMPC-PDPA

## Abstract

Mutations in *parkin* cause autosomal recessive Parkinsonism and mitochondrial defects. A recent drug screen identified a class of steroid-like hydrophobic compounds able to rescue mitochondrial function in *parkin-*mutant fibroblasts. Whilst these possess therapeutic potential, the size and high hydrophobicity of some may limit their ability to penetrate the blood-brain barrier from systemic circulation, something that could be improved by novel drug formulations. In the present study, the steroid-like compounds Ursolic Acid (UA) and Ursocholanic Acid (UCA) were successfully encapsulated within nanoscopic polymersomes formed by poly(2-(methacryloyloxy)ethyl phosphorylcholine)–poly(2-di-isopropylamino)ethyl methacrylate) (PMPC-PDPA) and separated into spherical and tubular morphologies to assess the effects of nanoparticle mediated delivery on drug efficacy. Following incubation with either morphology, *parkin-*mutant fibroblasts demonstrated time and concentration dependent increases in intracellular ATP levels, resembling those resulting from treatment with nascent UA and UCA formulated in 0.1% DMSO, as used in the original drug screen. Empty PMPC-PDPA polymersomes did not alter physiological measures related to mitochondrial function or induce cytotoxicity. In combination with other techniques such as ligand functionalisation, PMPC-PDPA nanoparticles of well-defined morphology may prove a promising platform for tailoring the pharmacokinetic profile and organ specific bio-distribution of highly hydrophobic compounds.

## Highlights

• Ursolic acid and ursocholanic acid are efficiently encapsulated within PMPC-PDPA polymersomes of spherical or tubular morphology.
• PMPC-PDPA polymersomes spherical or tubular morphology are found to enter into *parkin*-mutant fibroblasts causing no deleterious effects.
• ATP levels are increased in *parkin*-mutant fibroblasts after incubation with ursolic or ursocholanic acid loaded PMPC-PDPA polymersomes.
• The efficacy of ursolic acid and ursochocholanic acid loaded PMPC-PDPA nanoparticles is similar to DMSO based formulations of the same compounds.

## 1 Introduction

Parkinson’s disease (PD) is one of the most common neurodegenerative disorders, the prevalence of which is expected to increase in the coming years [1]. Clinical strategies currently offer symptomatic relief, no therapy to slow disease progression is presently available. Great efforts have been taken to identify small-molecules capable of rectifying the pathogenic processes implicated in PD, in the hope of producing a readily administrable disease modifying therapy.

Mitochondrial dysfunction has been observed in familial and sporadic forms of PD [2], [3]. In particular we and others have described mitochondrial dysfunction in fibroblasts derived from patients with *parkin* mutations, the most common identifiable cause of early onset, familial PD [4], [5]. We identified a number of compounds able to restore mitochondrial function in *parkin*-mutant patient fibroblasts [6]. Compounds of particular interest with structural similarities are Ursolic Acid (UA), Ursocholanic Acid (UCA), and Ursodeoxycholic Acid (UDCA), owing to their efficacy, availability, and existing knowledge of their pharmacokinetic profiles. In addition, we demonstrated that UCA and UDCA produced therapeutic effects in *parkin* knock-down mouse cortical neurons, and fibroblasts from patients with G2019S *LRRK2* mutations, the most common known cause of familial PD and a gene implicated in sporadic PD [7], [8]. Evidence elsewhere has demonstrated neuroprotective effects of UA [9], [10]. Therefore, these compounds have therapeutic potential in at least some forms of PD.

However, efficacy *in vivo* for any given compound depends on its ability to reach the target tissue at therapeutically effective concentrations. Diseases of the brain are notoriously difficult to treat owing to the presence of the blood-brain barrier (BBB), a hurdle that reportedly 98% of known small molecule therapeutics fail to effectively penetrate from systemic circulation [11], [12], [13]. Owing to the high hydrophobicity of both UA and UCA, penetrance across the BBB may be unfavourable following intravenous or oral administration due to poor solubility within blood [13]. Indeed, though UA has been found in the brains of rats following oral administration, the percentage distribution here was extremely small even after several months of continuous administration [14], notable, given the side effects that have been associated with excessive UA doses [15], [16]. The use of formulations that improve the circulation times and/or specificity for BBB interactions of these compounds, without modification to their existing chemistry are thus desirable.

The use of bio-compatible polymeric nanoparticles as alternatives to existing therapeutic drug formulations has received much attention given their synthetic and thus exceptionally tailorable natures [17], [18], [19]. They are formed from amphiphilic block copolymers that self assemble and are held together in water by weak interactions, facilitating efficient drug -encapsulation and importantly, -release. Yet the macromolecular nature of their component polymers makes them non-ergodic, i.e. once formed, a particle does do not exchange material with its environment, resulting in high stability. They possess dense outer coronas formed from charge neutral, water soluble chemistries that reduce interactions with plasma proteins so improving their circulation half-lives in blood [20], [21].

The size and shape of polymeric nanoparticles may also be modified for specific applications, the diameter of spherical particles is known to influence the rate and route of uptake into cells. Particle morphologies with increased aspect ratio show increased circulation half-lives in blood [22], [23], [24]. Shape also modifies nanoparticle binding affinities, by merit of the different shear stresses more or less streamlined particles experience in blood flow and alterationzs to surface areas that may effectively interact with a biological surface. Thus nanoparticles morphology may be used to enhance the targeting effects bestowed by the attachment of receptor-specific ligands to nanoparticles. Finally, in packaging a compound within a carrier and delivering them to cells as “payloads”, enhancements to therapeutic efficacy relative to the freely diffused molecule can be achieved both *in vitro* and *in vivo* [26], [27], [28].

Polymersomes are the macromolecular analogues to liposomes and indeed have been evaluated for several applications in drug delivery. Numerous reports have detailed the ability of Poly(2-(methacryloyloxy)ethylphosphorylcholine)–poly(2-diisopropylamino)ethyl methacrylate) (PMPC-PDPA) polymersomes to enter into a variety of primary and immortalised cells whilst causing no apparent inflammatory responses or cell toxicity [29], [30], [31], [32]. They have also been shown to encapsulate a range of hydrophilic and hydrophobic compounds, which then enter the cell and are released there via the endocytic route of entry and pH-sensitive disassembly of PMPC-PDPA polymersomes within the early endosome [30], [31], [32], [33]. It has been shown that LRP-1 targeting polymersomes successfully cross the BBB via transcytosis, wherein they deliver macromolecular cargoes to cells of the CNS [25]. We recently described a method to create and purify spherical and tubular PMPC-PDPA polymersomes, morphologies which demonstrate distinct biological behaviours that could be exploited for specific therapeutic applications, including enhancement of BBB specific interactions [22], [23], [34], [35].

Here we describe a proof of concept study in which UA and UCA are formulated within spherical and tubular PMPC-PDPA nanoparticles and their interactions with *parkin-*mutant fibroblasts are assessed. Should these demonstrate similar or improved therapeutic effects in *parkin-*mutant fibroblasts relative DMSO based formulations, whilst producing no-deleterious effects to viability and mitochondrial function, such particles may prove a promising platform for tailoring drug bio-distribution such as improved brain penetrance.

## 2 Materials and methods

### 2.1 Small molecules

UA, UCA and cholesterol were all purchased from Sigma-Aldrich Ltd. at the highest available purity. Dehydroergosterol (DHE) was purchased from Avanti polar lipids inc. Compounds were dissolved in ethanol or Dimethyl Sulfoxide (DMSO), for the purposes of nanoparticle encapsulation and cell culture respectively.

### 2.2 Block co-polymers

PMPC_25_-PDPA_65_ was synthesised by Dr. N. Warren as previously described [36] and Rhodamine-6G-PMPC_25_-PDPA_70_ by Dr. J. Madsen as previously described [37].

### 2.3 Nanoparticle formation, separation and characterisation

Nanoparticles were formed in PBS from thin-films of PMPC_25_-PDPA_65_ alone or pre-mixed with UA/UCA using the thin-film rehydration method (TFR), as described elsewhere [34]. Preparations were designed, assuming total efficiency of nanoparticle formation, to produce solutions containing 1 mM UA or UCA and 10 mg/mL PMPC_25_-PDPA_65_. The resulting nanoparticles were purified by hollow fibre filtration (HFF) (KrosFlo Research IIi, SpectrumLabs), using a 1 in 100 dilution and a polysulfone based separation module (500 kDa molecular weight cut-off). Nanoparticles were then separated into polymersome, tubular polymersomes, and lyotropic structure fractions by centrifugal fractionation at 2000 and 15,000 RCF, as previously described [34].

### 2.4 Dynamic light scattering

Particle size distributions were assessed by dynamic light scattering (DLS) (Zetasizer nano ZS, Malvern Ltd.) at copolymer concentration of 0.25 mg/mL as previously described [36].

### 2.5 Transmission electron microscopy

Nanoparticles were imaged by Transmission Electron Microscopy (TEM) (FEI Tecnai G2 Spirit electron microscope) and images captured on a Gatan Digital Micrograph at 120 keV. TEM samples were prepared as described elsewhere [36]. Briefly, nanoparticles were applied (1 mg/mLPMPC-PDPA) to glow discharged copper grids (Agar Scientific) coated with carbon, and stained with phosphotungstic acid (0.75% w/w).

### 2.6 High performance liquid chromatography

Polymer and encapsulate concentrations were determined by reverse-phase performance liquid chromatography (RP-HPLC). Nanoparticle samples were dried under vacuum overnight, and re-solubilised in a 4:1 mixture of acidified methanol (Chromasolv, Sigma-Aldrich) and Milli-Q filtered water, plus 0.1% Trifluoroacetic Acid (TFA). RP-HPLC was performed with a C18 analytical column (phenomenex Jupiter; octadecylsilyl ultra-pure silica, C18, 300°A, 150 × 4.6 mm, 5 μm) using methanol (0.1% TFA) and Milli-Q filtered water (0.1% TFA) as eluents A and B respectively in the following gradient (column temperature 30 °C, flow rate 1 mL/min); 0 min 30% eluent B, 5 min 30% eluent B, 12 min 100% eluent B, 22 min 100% eluent B, 23 min 30% eluent B, 28 min 30% eluent B. With use of standard curves, PMPC-PDPA, DHE and UA concentrations were determined by UV detection (205 nm), and UCA concentrations by evaporative light-scattering detection (ELSD). Work involving UV detection alone was performed on Dionex ultimate 3000 system (MWD-3000 UV detector), and analysed using Chromeleon software (Dionex). Work requiring ELSD was performed on a Shimadzu UFLC XR system (SPD-M20A diode array detector, Alltech ELSD 800), and analysed with Labsolutions LCSolution software (Shimadzu).

### 2.7 Patient details

Punch skin biopsies were taken from three patients with compound heterozygous mutations in *parkin*, following routine clinical procedures (mean age 35 years old ± 2.46 SD) [4]. Patient PD1 has het202-203delAG(exon2)/hetExon2del, patient PD2 has het202-203delAG(exon2)/hetExon4del and patient PD3 has c.101-102delAG(exon1)/c.1289G > A(exon11). These were paired with control fibroblasts obtained from three healthy controls age and sex matched (mean age 36.7 years old ±3.21 SD, obtained from the NIGMS Human Genetic Cell Repository at the Coriell Institute for Medical Research: GM08400, GM08402, GM07300)

### 2.8 Cell culture

Primary fibroblast cells were cultured continuously in glucose media (Minimum Essential Medium with 10% FBS, 100 IU/ml penicillin, 100 μg/mL streptomycin, 1 mM sodium pyruvate, 2 mM l-glutamine, 0.1 mM amino acids, 50 μg/mL uridine and 1 X MEM vitamins). Control and patient fibroblasts were used between passages 11 and 14, matched to within one passage of each other.

For physiological assays, fibroblasts were grown in glucose media for 24 h, then switched into a galactose media (DMEM without glucose, supplemented with 10% FBS, sodium pyruvate 1 mM, penicillin 100 IU/mL, streptomycin 100 μg/mL and galactose 0.9 mg/mL), as described before [4]. Fibroblasts were cultured in galactose for 24 h before addition of experimental conditions.

### 2.9 Intracellular ATP levels

Cellular Adenosine Triphosphate (ATP) levels were measured using the ATPlite kit (PerkinElmer) as described previously [4].

### 2.10 LDH release

Cytotoxic induction was assessed using the CytotoxOne homogeneous membrane integrity kit (Promega) according to the manufacturer’s instructions.

### 2.11 TMRM assay

Mitochondrial membrane potential (MMP) was assessed using a TMRM assay adapted for use in a 96 well plate format, as described elsewhere [4].

### 2.12 Flow cytometry

Uptake of Rhodamine and/or DHE labelled PMPC-PDPA nanoparticles was assessed by flow cytometry as previously described [32], [34]. Results show median fluorescent intensities (MFI) from 10,000 fibroblasts as determined by FSC and SSC gating, minus autofluorescence as measured in untreated fibroblasts.

### 2.13 Statistical analysis

Statistical significance of single experimental factors was assessed using one-way ANOVA with Bonferoni corrected multiple comparisons. Statistical significance of two experimental factors was assessed by two-way ANOVA with Bonferoni corrected multiple comparisons.

## 3 Results

### 3.1 Characterisation of drug loaded PMPC-PDPA nanoparticles

**Fig. 1:**
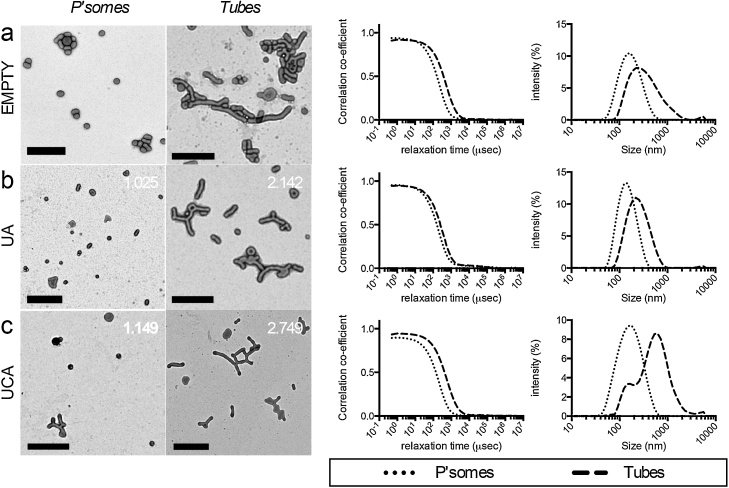
UA and UCA loaded nanoparticles. From left to right, TEM images of polymersome and tube fractions (scale bars = 500 nm, mol/mol cargo:copolymer ratios indicated), DLS auto-correlation functions and estimated size distribution by intensity from (a) empty, (b) UA and (c) UCA loaded nanoparticles.

Four weeks formation by TFR produced nanoparticle populations of mixed morphology [34]. High drug encapsulation efficiencies were seen with little decrease in drug to polymer ratio following purification of free drug from the mixed morphologies (Table 1), indicating that the majority of both UA and UCA were successfully incorporated into the polymersomes. Purified PMPC_25_-PDPA_65_ nanoparticles were centrifugally fractionated to form separate populations enriched with spherical or tubular polymersomes, henceforth referred to as polymersomes and tubular polymersomes respectively (Fig. 1). Interestingly, drug-polymer quantification revealed a greater concentration of UA and UCA relative to PMPC_25_-PDPA_65_ present within the tubular fractions (Table 1), and that the relative proportion of tubular polymersome increased with UA or UCA load, in keeping with a previous report [34].

### 3.2 Vesicular PMPC-PDPA nanoparticles cause no cellular toxicity

**Fig. 2:**
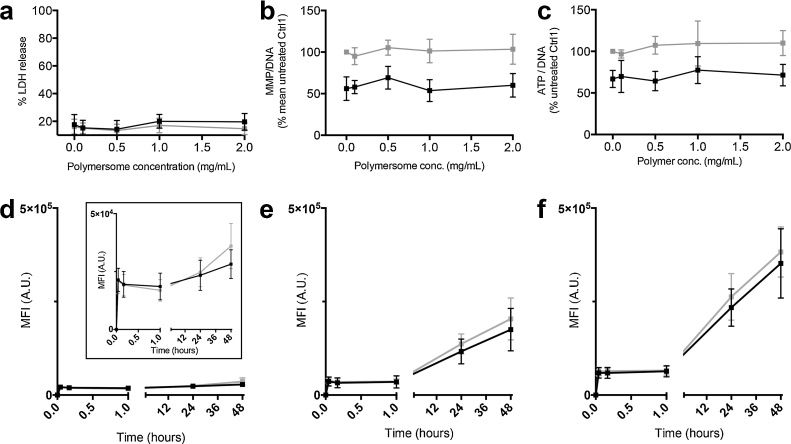
PMPC_25_-PDPA_65_ nanoparticles of mixed tubular and polymersome morphologies enter, and cause no apparent toxicity to, *parkin-*mutant fibroblasts. (a) Percentage LDH release, (b) cellular ATP levels and (c) MMP were assessed in a set of age matched *parkin-*mutant (black) and control (grey) fibroblasts following 48 h incubation with PMPC_25_-PDPA_65_ of nanoparticles of mixed morphology (error bars = SD, n = 3). The uptake kinetics of Rh.6G labelled PMPC_25_-PDPA_65_ nanoparticles of mixed morphology was assessed by flow cytometry at concentrations of (d) 0.1 (inset shows results on a reduced MFI scale), (e) 0.5 and (f) 1 mg/mL (mean MFIs from three sets of age matched *parkin-*mutant and control fibroblasts shown, error bars = SD, n = 3).

Following 48 h incubation with mixed morphology nanoparticles (0, 0.1, 0.5, 1 or 2 mg/mL), *parkin-*mutant and control fibroblasts demonstrated no apparent cytotoxicity (Fig. 2A). Furthermore, no alteration to MMP (Fig. 2B) or cellular ATP levels (Fig. 2C) were detected at these concentrations. The previously described defects in *parkin-*mutant fibroblast mitochondrial function were also seen (expressed as %Ctrl1 ± SD, PD1 baseline MMP = 55.6 ± 11.9, and ATP = 65.6 ± 4.4) [4]. Similar results were noted in a second set of *parkin-*mutant fibroblasts and matched control (Supp. Fig. 1). Incubation with separated polymersomes or tubular polymersomes also had no effect on the cellular ATP levels of control or *parkin*-mutant fibroblasts (Supp. Fig. 2).

### 3.3 Uptake of nanoparticles into parkin mutant fibroblasts

The kinetics of nanoparticle uptake in fibroblasts was investigated by flow cytometry in three primary *parkin*-mutant fibroblast lines plus matching controls, incubated with mixed morphology nanoparticles. A rapid nanoparticle binding step was seen within the first minute of incubation followed by a linear phase of more gradual nanoparticle internalisation over the next 48 h, both of which were proportional to nanoparticle concentration (Fig. 2D–F). No significant difference in nanoparticle uptake was seen between the fibroblast phenotypes as groups, though a deficiency in one *parkin-*mutant fibroblast relative to controls was noted.

In order to assess the uptake of separated polymersomes and tubular polymersomes, as well as their ability to deliver steroid-like cargoes, further experiments using separated Rh.6G-labbelled PMPC_25_-PDPA_65_ polymersomes and tubular polymersomes loaded with dehydroergosterol (DHE), a naturally occurring fluorescent sterol, were performed. Polymersome accumulation into fibroblasts was greater than that of tubular polymersomes following both 24 and 48 h incubation (Supp. Fig. 3). Polymersomes also delivered greater DHE loads, however to a much lesser extent than the entry of the nanoparticles themselves, likely reflecting the greater DHE concentrations found in tubular polymersomes relative to polymersomes (DHE:PMPC_25_-PDPA_65_ mol/mol ratios for tubular polymersomes = 2.689, for polymersomes = 1.164 respectively).

### 3.4 Therapeutic efficacy of UA and UCA loaded, vesicular PMPC-PDPA nanoparticles

**Fig. 3:**
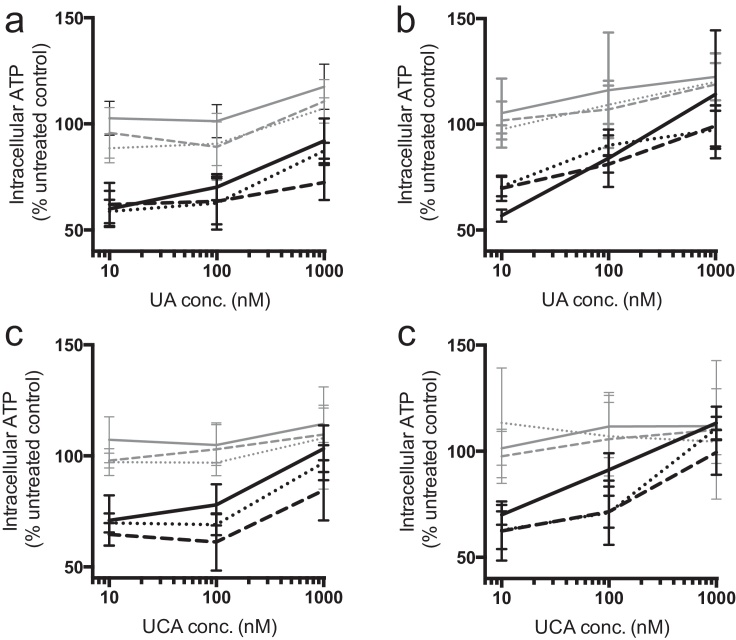
UA and UCA encapsulated within PMPC_25_-PDPA_65_ polymersomes (dotted lines) and tubes (dashed lines), elicit dose dependent increases in fibroblast ATP levels similar to when dissolved in 0.1% DMSO (solid lines). Control (grey) and matched *parkin-*mutant fibroblasts (black) were incubated with formulations of UA for (a) 24 and (b) 48 h, as well as UCA formulations for (c) 24 and (d) 48 h (error bars = SD, n = 3).

The effect of empty and UA/UCA loaded polymersomes and tubular polymersomes on intracellular ATP levels were assessed in two sets of matched *parkin-*mutant and control fibroblasts, following 24 and 48 h incubation. Separated morphology nanoparticles were applied to treat cells with 10, 100 or 1000 nM UA or UCA or at matching copolymer concentrations of empty nanoparticles. Equivalent concentrations of UA/UCA dissolved in DMSO were also tested. Following incubation with UA or UCA loaded nanoparticles, *parkin*-mutant fibroblasts showed increases in intracellular ATP levels similar in magnitude to those induced by DMSO based formulations (Fig. 3 and Supp. Table 1), concentrations of 1000 nM UA or UCA, significant increases in *parkin*-mutant fibroblast ATP levels (p < 0.05) were reached by all three drug formulations, with the exception of UA/UCA loaded tubular polymersomes following 24 h incubation (Supp. Table 1). Tube based formulations appeared less efficacious following 24 h incubation (Fig. 3A, C), and DMSO based formulations produced larger mean ATP levels, with one exception (UCA DMSO vs UCA tubular polymersomes following 48 h incubation in *parkin*-mutant fibroblasts p < 0.05) no significant differences between formulations were detected at either time-point, within the fibroblast phenotypes. Overall, a significant dependence on the presence of *parkin* mutations for ATP recovery from baseline within each UA and UCA formulations was seen (p < 0.001 in all instances), as were significant effects of UA and UCA formulation concentrations within *parkin-*mutant fibroblast ATP levels (p < 0.001 in all instances). Unloaded nanoparticles had no significant effect on fibroblast ATP levels (Supp. Fig. 2).

## 4 Discussion

Both UA and UCA were efficiently encapsulated within vesicular PMPC_25_-PDPA_65_ nanoparticles using the TFR methodology. Purification by HFF resulted in little change in drug to copolymer ratios indicating very little drug escaped encapsulation, likely owing to the very high hydrophobicity of both UA and UCA. Empty vesicular PMPC_25_-PDPA_65_ nanoparticles of mixed morphology were found to enter *parkin-*mutant fibroblasts whilst inducing no apparent changes to cell membrane integrity, mitochondrial membrane potential or intracellular ATP levels.Separated polymersomes and tubular polymersomes had no effect on cellular ATP levels. Having shown no overt deleterious effects to *parkin-*mutant fibroblast viability or mitochondrial function in the face of existing physiological defects, PMPC_25_-PDPA_65_ nanoparticles show promise as therapeutic vectors for *parkin-*mutant fibroblasts, and perhaps other cells possessing known mitochondrial defects [5], [6]

Vesicular PMPC_25_-PDPA_65_ nanoparticles entered fibroblasts linearly over 48 h after an initial rapid binding phase, both of which were proportional to copolymer concentration. No significant differences in uptake were found between control and *parkin-*mutant cells. This is perhaps contradictory to work from Sack and co-workers in which *parkin*-mutant cells demonstrated reduced lipid uptake as a result of altered class B scavenger receptor expression, the same class of receptors implicated in the uptake of PMPC-PDPA nanoparticles [27], [38]. This may arise from differences in the size, morphology, and chemistry of the particles investigated in each study as well as the different cell in which their uptake was assessed. In addition, the two studies investigated different *parkin* mutations: where all cell models tested by Sack and co-workers possessed exon 2 mutations in both *parkin* alleles, only one of the primary fibroblast lines used in the present study shared this genotype [38]. These same fibroblasts were also the only to demonstrate a clear deficiency in nanoparticle uptake relative to controls. Since exon 2 of Parkin has been linked to the regulation of Class B scavenger receptors, it may be that its complete loss of function is necessary for reduced lipid and PMPC-PDPA nanoparticle uptake [38], [39].

Tubular polymersomes were found to enter into fibroblasts less readily than polymersomes, in keeping with studies comparing the cellular uptake of similar nanoparticle morphologies [22], [34], [35]. Their ability to deliver steroidal cargo however was more closely matched. This is likely due to the greater cargo to copolymer ratios found in the tubular fraction of PMPC_25_-PDPA_65_ nanoparticles, wherein a single tube will hold and deliver a greater cargo concentration than an equivalent mass of polymersomes.

Both UA and UCA loaded nanoparticles produced concentration and time dependent increases in the cellular ATP levels of *parkin*-mutant fibroblasts with a similar efficacy to DMSO based formulations. Though not significant, a lower mean efficacy was seen from tubular polymersomes relative to polymersomes following 24 h incubation, which may be linked to the slower uptake of tubular polymersomes. This could also result from the at least partially different routes of entry tubular polymersomes and polymersomes take into the cell [34].

While drug encapsulation within nanoparticles has been noted to decrease drug efficacy in at least one instance [40], the majority of previous reports show improved drug efficacy when compared to “free drug” formulations. This has been attributed to nanoparticles’ ability to deliver drug “pay loads” to the cell, transiently creating higher local drug concentrations than could be achieved by simple diffusion [26], [27]. For instance, UA loaded in methoxy-poly(ethylene glycol)–polycaprolactone (mPEG–PCL) nanoparticles have shown enhanced cytotoxicity against gastric and liver cancer cell lines [28], [41]. UA nano-suspensions and nano-crystals have also demonstrated enhanced therapeutic effects over UA in solution [42], [43]. That efficacy was not improved in the present study may be attributable to a number of factors, including the relatively poor uptake of PMPC-PDPA polymersomes in fibroblasts relative to many other cell types and the use of galactose- instead of glucose- cell media, which results in lowered PMPC-PDPA polymersome uptake (Supp. Fig. 4) [27], [32].

Route of delivery may also impact on drug efficacy. Co-solvation in DMSO will allow UA and UCA to interact directly with the cell membrane, as well as adsorb to serum proteins and tissue culture plastics. Encapsulation within PMPC-PDPA nanoparticles on the other hand shields against these extracellular interactions and results in direct delivery of compounds into the cell. That DMSO based formulations produced somewhat more marked increases in mean cellular ATP levels might suggest that, though intracellular delivery UA and UCA is effective, external interactions with the cell may be more efficacious. Our previous work showed that the rescue effect of DMSO formulated UCA was mediated via the glucocorticoid receptor and Akt signalling [6], however whether these pathways are utilised following direct delivery into the cell remains to be investigated.

The high drug loading and slower internalisation kinetics of tubular polymersomes may make these of particular use for therapeutic applications. *In vivo,* improvements to a compounds therapeutic potential when packaged within nanoparticles will rely, in part, on enhancements to their target specific delivery. Tubular morphologies have been shown to experience greater shear stresses within fluid flow [24], [44] and to circulate in the blood of mice for durations far exceeding any other tested morphology [22]. When functionalised with receptor ligands, such particles also demonstrate greater specificities to their target tissues *in vivo* [23], [24]. Discher and co-workers suggested a paradigm which uses tailored receptor ligands of high and low affinity, and particle morphologies that experience well defined shear stresses in flow, may allow for highly specific targeting of particles to endothelial walls, even where unique receptor targets do not exist, such as is the case in the BBB [23]. In combination with strategies to facilitate the passage of nanoparticles across the BBB, such as recently described for Angiopep-2 functionalised PMPC-PDPA polymersomes, tubular polymersomes have the potential to enhance the brain specific bio-distribution of small, highly hydrophobic cargoes, such as UA and UCA [25].

## 5 Conclusion

UA and UCA loaded PMPC_25_-PDPA_65_ nanoparticles were successfully formed with high encapsulation efficiencies, and spherical or tubular polymersome morphologies isolated from these. Both demonstrated an ability to increase the cellular ATP levels of *parkin-*mutant fibroblasts, similar to DMSO based formulations, without any apparent cytotoxicity or detriment to mitochondrial function. Thus encapsulation of UA and UCA in PMPC_25_-PDPA_65_ polymersomes or tubular polymersomes holds promise for the treatment of *parkin*-mutant cells. In combination with other techniques such as ligand functionalisation, one may be able to tailor the pharmacokinetic profiles of such particles to enhance the brain specific distributions of UA and UCA.

## Conflicts of interest

The authors declare no competing financial or personal interests.

## Contributors

G.Y. conducted majority of the experiments. G.Y. and H.M. performed ATP assays. H.M, G.B. and O.B. contributed to hypothesis formulation. G.B. and O.B. contributed to the overall project supervision. All authors contributed to the writing of the manuscript.

## Figures and Tables

**Table 1:** Encapsulation efficiencies of UA and UCA loaded PMPC_25_-PDPA_65_ mixed morphology nanoparticles (±SD), pre- and post- purification by HFF.

		PMPC_25_-PDPA_65_ (mg/mL)	Drug (mg/mL)	Drug encapsulation efficiency	Drug:PMPC-PDPA (mol/mol)
	*Initial conc.*	10	1000	100	2.12
*Pre-HFF*	*Empty*	7.8 ± 0.42	–	–	–
	*UA*	8 ± 0.71	667.6 ± 4.9	66.7	1.754
	*UCA*	8.6 ± 0.7	703.2 ± 7.1	70.32	1.719

*Post-HFF*	*Empty*	5.8 ± 0.93	–	–	–
	*UA*	5.3 ± 0.75	392.7 ± 3.2	39.27	1.549
	*UCA*	5.9 ± 0.24	442.5 ± 4.5	44.25	1.584
