# Supplementary material for: Rescue of mitochondrial function in parkin-mutant fibroblasts using drug loaded PMPC-PDPA polymersomes and tubular polymersomes
Source: Neurosci Lett. 2016 Sep 6;630:23–9. doi: 10.1016/j.neulet.2016.06.065 (PMC5010038; doi:10.1016/j.neulet.2016.06.065)
Supplement: Supplementary file 1 [file mmc1.pdf]

Supplemental Material: Rescue of mitochondrial function in *parkin*-mutant Fibroblasts using drug loaded PMPC-PDPA polymersomes and tubular polymersomes

G. Yealland[a,b,] G Battaglia[c], O. Bandmann O[b], H. Mortiboys H[b*]
[a]Biomedical Sciences, University of Sheffield, Firth Court, Western Bank, Sheffield S10 2TN
[b]SITraN, Neuroscience, University of Sheffield, 385a Glossop Road, Sheffield S10 2HQ
[c]Department of Chemistry, UCL, 20 Gordon Street, London WC1H 0AJ
*** Corresponding author** H.Mortiboys@sheffield.ac.uk

Supplemental Results

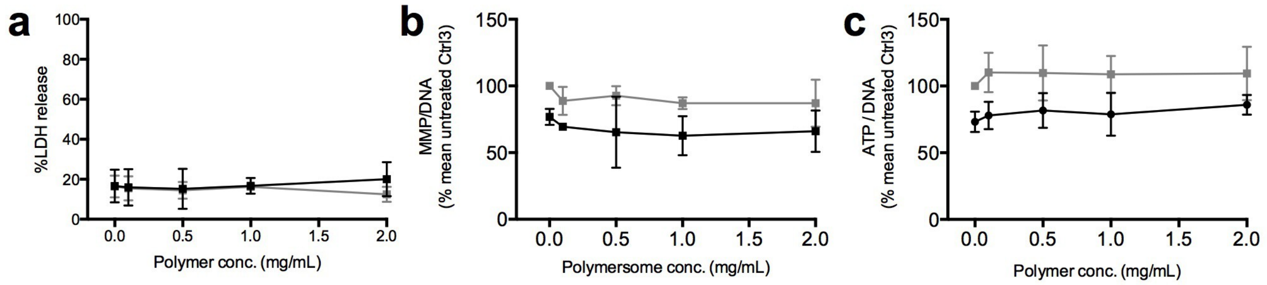

Supplemental figure 1: $PMPC_{25}$-$PDPA_{65}$ nanoparticles of mixed tubular and polymersome morphologies enter, and cause no apparent toxicity in a second set of age matched *parkin*-mutant (black) and control fibroblasts (grey). (a) Percentage LDH release, (b) cellular ATP levels and (c) MMP following 48 hour incubation with $PMPC_{25}$-$PDPA_{65}$ of nanoparticles of mixed morphology (error bars = SD, n = 3).

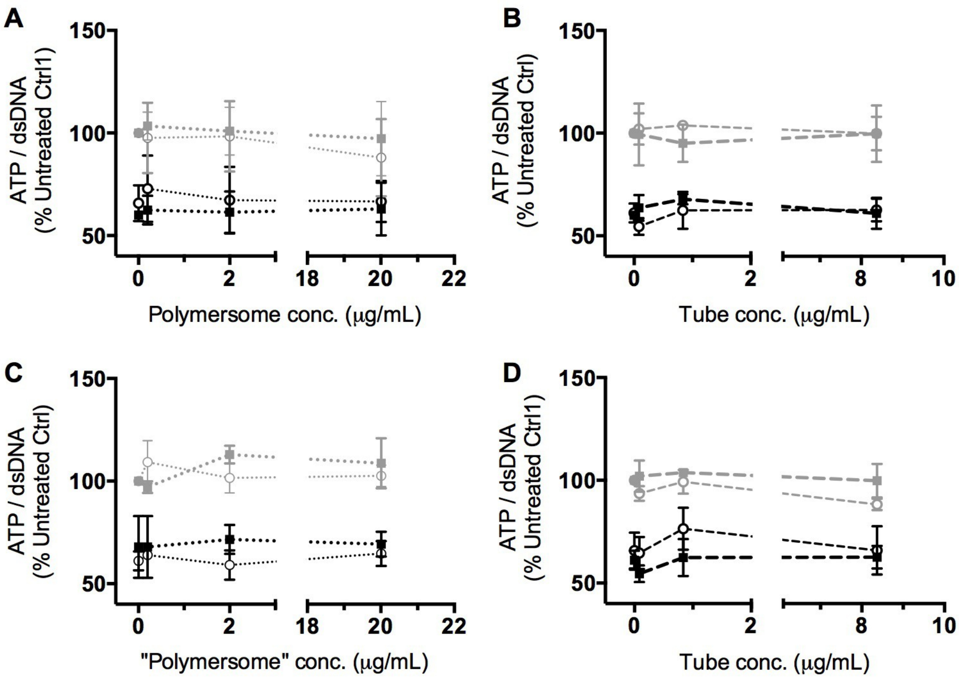

Supplemental figure 2: $PMPC_{25}$-$PDPA_{65}$ Polymersome and Tube fractions cause no apparent change to *parkin*-mutant or wild-type fibroblast cellular ATP levels. (a, b) Matched control and *parkin*-mutant fibroblasts were incubated with polymersomes (dots) or tubes (dashes) for 24 (open circles) and 48 (closed squares) hours at copolymer concentrations equivalent to UCA loaded nanoparticles incubated with cells at UCA concentrations of 10, 100 and 1000nM. (c, d) A second set of matched control and *parkin*-mutant fibroblasts were also tested (error bars = SD, n = 3).

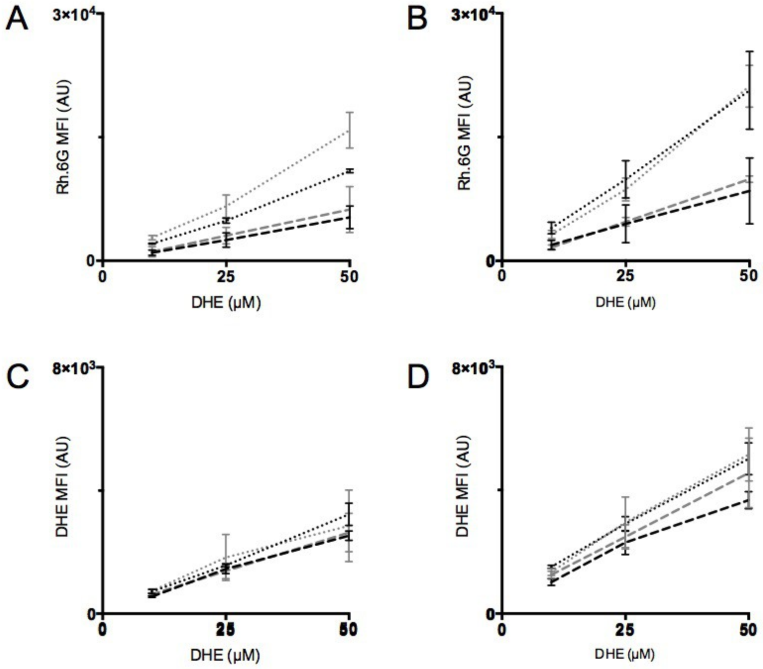

Supplemental figure 3: Uptake of separated, DHE loaded, $PMPC_{25}$-$PDPA_{65}$ polymersomes and tubes. Control (grey) and *parkin*-mutant (black) fibroblasts were incubated with DHE loaded polymersomes (dotted lines) and tubes (dashed lines) at cargo matched concentrations for (a, c) 24 and (b, d) 48 hours. (a, b) Rh.6G-PMPC-PDPA and (c, d) DHE MFIs were assessed by flow cytometry (error bars = SD, n = 3).

Supplemental table 1: Mean intracellular ATP levels (±SD) of two *parkin*-mutant fibroblast primary lines following treatment with UA (a) or UCA (b) formulations. Results are expressed as the percentage of ATP found in control fibroblasts (age-sex matched). Statistically significant increases in ATP levels, relative to untreated *parkin*-mutant fibroblasts, are indicated in blue ($n = 3$, statistical significance calculated by two way ANOVA with Bonferoni corrected multiple comparisons; * = $p < 0.5$, ** = $p < 0.1$, *** = $p < 0.001$, **** $p < 0.0001$)

		PD1		PD2	
	Drug Conc.	24hr	48hr	24hr	48hr
**a** UA formulated in P'somes	0	60.7±10.9	62.1±4.1	61.1±4.6	60.9±6.5
	10	58.7±5.5	70.6±6.3	69.0±7.3	63.2±4.1
	100	62.7±12.4	*** 90.1±5.2	80.8±6.5	76.7±3.5
	1000	** 87.3±3.8	**** 97.4±9.7	**** 96.5±6.5	*** 90.1±5.5
UA formulated in Tubes	0	60.7±10.9	62.1±4.1	61.1±4.6	60.9±6.5
	10	62.1±10.2	69.78±6.0	63.4±7.5	63.9±6.9
	100	63.5±9.1	* 81.1±4.0	70.6±6.4	70.0±5.4
	1000	72.4±8.2	**** 99.3±9.7	79.7±8.4	** 89.0±9.1
UA formulated in 0.1% DMSO	0	60.7±10.9	62.1±4.1	61.1±4.6	60.9±6.5
	10	59.9±8.6	56.78±2.9	62.6±6.8	59.7±4.8
	100	70.2±6.2	84.0±13.6	76.0±9.2	79.5±3.2
	1000	**** 92.0±10.6	**** 114.2±30.3	**** 102.4±5.7	*** 91.6±2.6
**b** UCA formulated in P'somes	0	65.8±8.7	60±2.4	65.5±16.3	61.1±4.6
	10	69.8±4.4	62.8±8.9	68.3±7.7	69.0±7.3
	100	69.0±4.7	71.0±15.2	80.8±5.1	80.8±6.5
	1000	**** 96.9±7.9	**** 110.7±5.5	* 86.4±8.7	**** 109.0±11.8
UCA formulated in Tubes	0	65.8±8.7	60±2.4	65.52±16.3	61.1±4.6
	10	64.6±5.11	62.4±14.0	69.0±7.3	63.4±7.5
	100	61.2±12.9	71.5±7.5	70.8±3.3	** 91.9±28.1
	1000	84.5±13.6	**** 99.4±10.6	*** 94.9±12.8	**** 110.1±7.2
UCA formulated in 0.1% DMSO	0	65.8±8.7	60±2.4	65.52±16.3	61.1±4.6
	10	70.9±11.3	70.0±6.7	74.7±20.0	63.2±6.6
	100	77.9±9.3	** 91.1±7.9	* 85.6±9.2	76.0±9.2
	1000	**** 103.2±10.5	**** 113.93±9.6	**** 105.3±2.5	**** 120.7±4.7

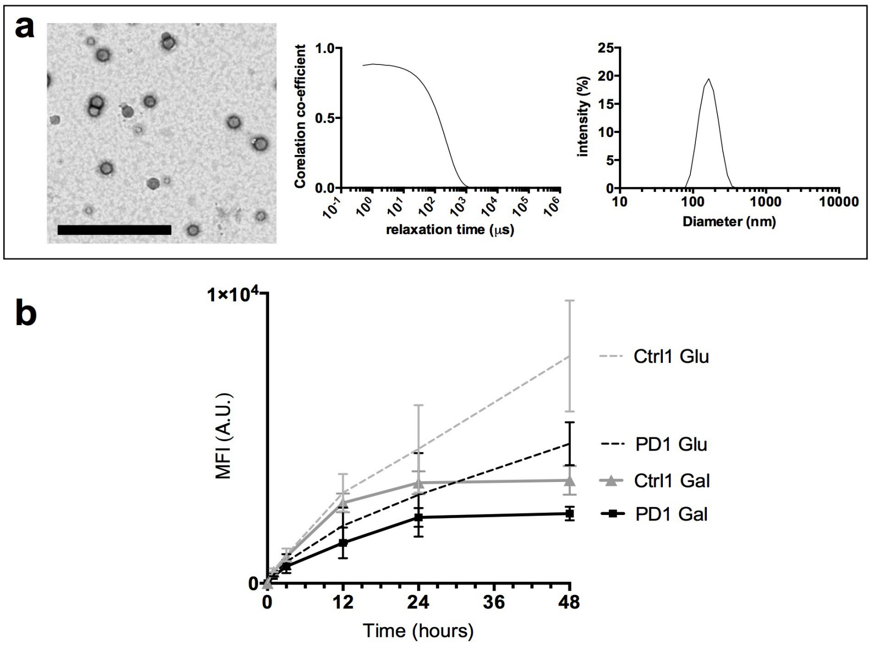

Supplemental Figure 4: Polymersome uptake is reduced when human dermal fibroblasts are cultured in galactose media. (a) Representative EM image (scale bar = 500nm), light scattering auto-correlation function and size distribution by intensity of $PMPC_{25}$-$PDPA_{70}$ polymersomes produced by pH switch. (b) Mean MFI of Rh.6G-PMPC-PDA following polymersome incubation with control or *parkin*-mutant fibroblasts (these uptake experiments done in 1 control and 1 *parkin*-mutant fibroblast line; error bars = SD, n = 3)
